# Hydatids of the Liver Evacuated Externally

**Published:** 1852-02

**Authors:** 


*Hydatids of the Liver Evacuated Externally.*—By M. Lafforgue.—A young female, after running rapidly down a hill, was seized with acute pain in the right hypochondrium. The ordinary signs of diseased liver soon supervened, and, notwithstanding the most varied modes of treatment, continued for five years. Her relations, believing that marriage would improve her condition, permitted her, in opposition to the advice of her physicians, to take a husband. The symptoms continued, though the disease did not appear to make progress. Six months after marriage she became pregnant. The symptoms now underwent exacerbation, but were soon mitigated, and the pregnancy advanced to the full term. The labor was natural, and the patient even nursed her child successfully. Finally, after the lapse of a year, her condition became more alarming, and M. Lafforgue was sent for. She was then in the following state:—Extreme emaciation, general blanching, excessive feebleness, impossibility of retaining food, which was rejected by vomiting almost as soon as swallowed, considerable tumefaction of right hypochondrium, descending to the crest of the ilium and transgressing the linea alba, obstructed respiration, orthopnœa. The respiration soon became more and more embarrassed, threatened with immediate asphyxia, the patient uttered stifled cries, demanded air, and seemed at the point of death. Lafforgue, detecting fluctuation in the tumor which compressed the lungs, made with the bistoury, between the eighth and ninth ribs, an opening which permitted the escape of a flood of thick serum. But the incision soon becoming obliterated by the presence of a thick granular matter, it became necessary to enlarge it, and immediately a hydatid, as large as a pear, was evacuated. The discharge was then re-established, bringing along with it hydatid sacs, of various sizes, some full, others completely empty, and with obliterated cavities; those which were so large as to stick in the wound were drawn out with forceps. In the space of a few minutes a vase, capable of holding six pounds of fluid, was filled with hydatids, and with a yellowish serosity of so fetid an odor as to annoy all the assistants.

The patient, after this enormous evacuation, fell into a state of extreme feebleness, the discharge was consequently arrested for the moment. Next day, however, uneasiness and dyspnœa were renewed, the bandage was removed, and a considerable additional quantity of hydatids and of fluid was evacuated. For six days the discharge continued spontaneously, and such was its abundance that it was estimated at twelve to fourteen pounds of fluid, containing several hundred hydatids. Gradually the symptoms disappeared, hydatids ceased to issue from the wound, and the fistula had nearly cicatrised, when the supervention of pains and uneasiness seemed to threaten a relapse. It proved, however, to be a second pregnancy, which led (by the pressure of the uterus) to the complete cicatrisation of the wound.—*Edinburgh Journal, from Journ. de Médecine et de Chirurgie Pratiques.*

